# From gut microbiota to host appetite: gut microbiota-derived metabolites as key regulators

**DOI:** 10.1186/s40168-021-01093-y

**Published:** 2021-07-20

**Authors:** Hui Han, Bao Yi, Ruqing Zhong, Mengyu Wang, Shunfen Zhang, Jie Ma, Yulong Yin, Jie Yin, Liang Chen, Hongfu Zhang

**Affiliations:** 1 State Key Laboratory of Animal Nutrition, Institute of Animal Science, Chinese Academy of Agricultural Sciences, Beijing, 100193 China; 2 Precision Livestock and Nutrition Unit, Gembloux Agro-Bio Tech, University of Liège, Passage de Déportés 2, 5030 Gembloux, Belgium; 3 College of Animal Science and Technology, Hunan Agricultural University, Changsha, 410128 China; 4 Key Laboratory of Agro-Ecological Processes in Subtropical Region, Institute of Subtropical Agriculture, Chinese Academy of Sciences, Changsha, Hunan 410125 China

**Keywords:** Gut microbiota, Appetite, Metabolites, Hormone, Immune

## Abstract

Feelings of hunger and satiety are the key determinants for maintaining the life of humans and animals. Disturbed appetite control may disrupt the metabolic health of the host and cause various metabolic disorders. A variety of factors have been implicated in appetite control, including gut microbiota, which develop the intricate interactions to manipulate the metabolic requirements and hedonic feelings. Gut microbial metabolites and components act as appetite-related signaling molecules to regulate appetite-related hormone secretion and the immune system, or act directly on hypothalamic neurons. Herein, we summarize the effects of gut microbiota on host appetite and consider the potential molecular mechanisms. Furthermore, we propose that the manipulation of gut microbiota represents a clinical therapeutic potential for lessening the development and consequence of appetite-related disorders.

**Video abstract**

**Supplementary Information:**

The online version contains supplementary material available at 10.1186/s40168-021-01093-y.

## Introduction

Feelings of hunger and satiety are principal involuntary motivations for feeding behavior in humans and animals [1–4]. Appetite, governed by the central nervous system (CNS), corresponds to a short-term signal from gastrointestinal hormones to control food intake and a long-term signal from adipose tissue associated with energy stores and environmental cues [5]. The CNS, hormones, and vagal afferents develop an intricate appetite system to initiate or inhibit food intake, while lack of physiological control of appetite generally results in eating disorders, such as anorexia nervosa (AN) and bulimia nervosa (BN), as well as metabolic diseases, such as obesity, which are potential threats to human host health [6–9].

Gastrointestinal tract is home to microbiota, which mutually interact with the host to modulate gut physiology and extraintestinal functions. Energy metabolism serves as a key point for microbiota and host interaction, as the gut microbiota not only receive energy from the host to maintain normal growth, but also supply the host with energy by releasing enzymes and metabolites, such as short-chain fatty acids (SCFAs), amino acids, bile acids (BAs), caseinolytic proteasB (ClpB), and lipopolysaccharide (LPS) [10]. To date, numerous studies are supporting the notion that gut microbiomes exert a profound influence on eating behavior in humans and animals [11–17]. Firstly, eating disorders are accompanied with alterations of gut microbiota. For example, AN patients have lower fecal microbial α-diversity and different fecal bacterial compositions [11, 18–20], while BN patients are characterized by a higher abundance of bacterial ClpB protein [21]. Secondly, gut microbial alterations further affect appetite and feeding behavior evidenced by a piglet model that lysine restriction-shaped microbial communities are associated with decreased circulating satiety hormones and increased feed intake [22]. Together, the gut microbiota and appetite system are highly associated and energy metabolism and microbial metabolites may serve as the potential mechanisms. Indeed, a review in 2017 has expertly summarized the integrative homeostatic model of appetite control related to the gut microbial metabolites mediated by bacterial growth cycle [10]. Diet interventions may dominate over host genetics to influence the incidence and development of metabolic diseases [23, 24], which is, at least in part, due to the modulations of gut microbial communities and metabolism. Thus, understanding the effects of nutrient-altered microbial metabolites on host metabolism and the potential molecular mechanisms provide an opportunity for the application of dietary interventions in metabolic diseases.

**Fig. 1:**
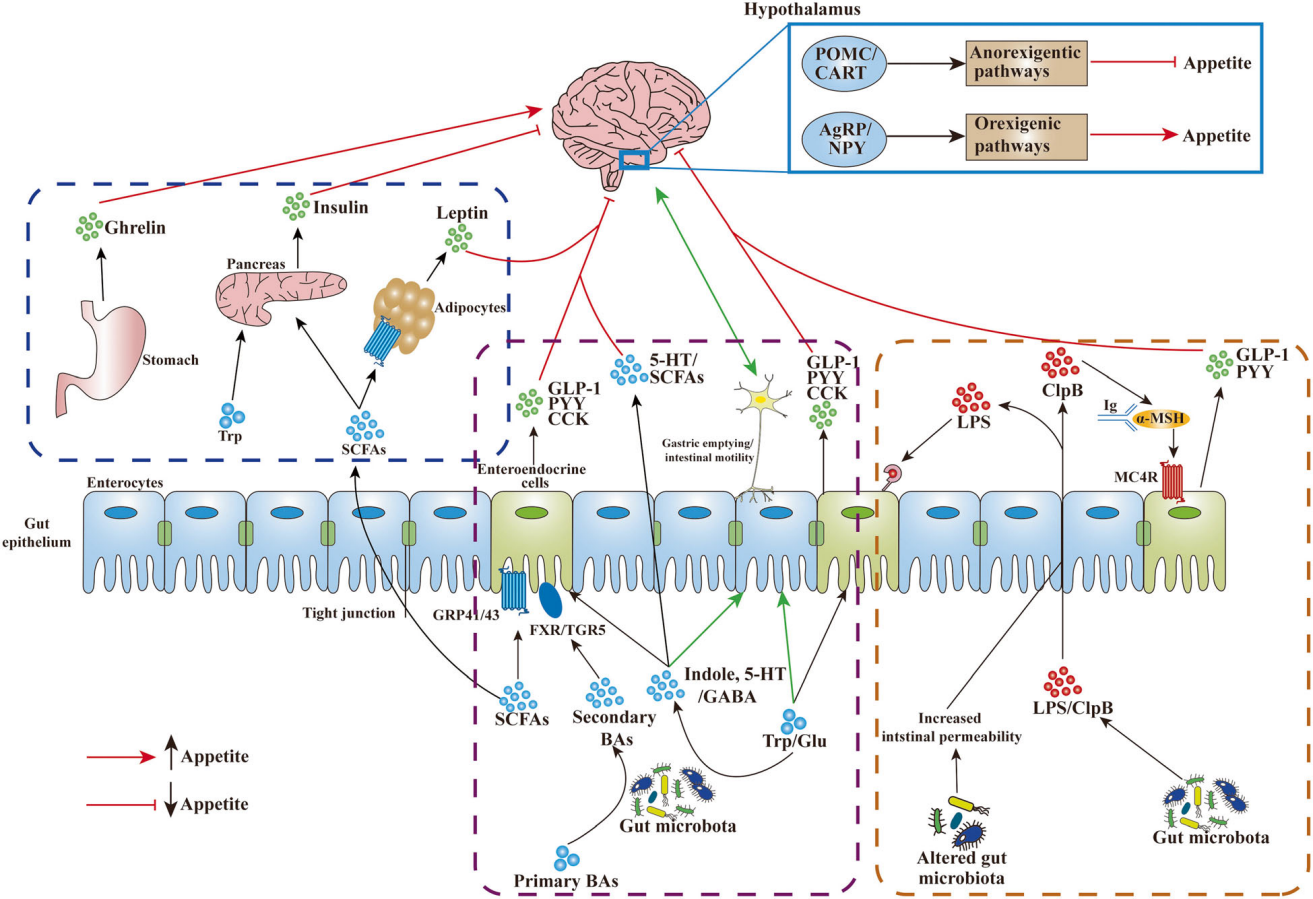
Gut microbiota-associated mechanisms involved in host appetite control. Firstly, gut microbial metabolites can stimulate enteroendocrine cells to release anorexigenic hormones (PYY, GLP-1, and CCK) and neurotransmitter (5-HT) and promote the secretion of peripheral hormones (leptin, ghrelin, and insulin). Secondly, Igs are involved in modulating the biological activity of appetite-regulating hormones, such as leptin and ghrelin. In addition, gut microbiota can produce identical protein sequences with appetite-regulating peptides, such as ClpB, that might directly act on anorexigenic neurons or bind to Igs to modulate the secretion of anorexigenic hormones from enteroendocrine L cells

**Fig. 2:**
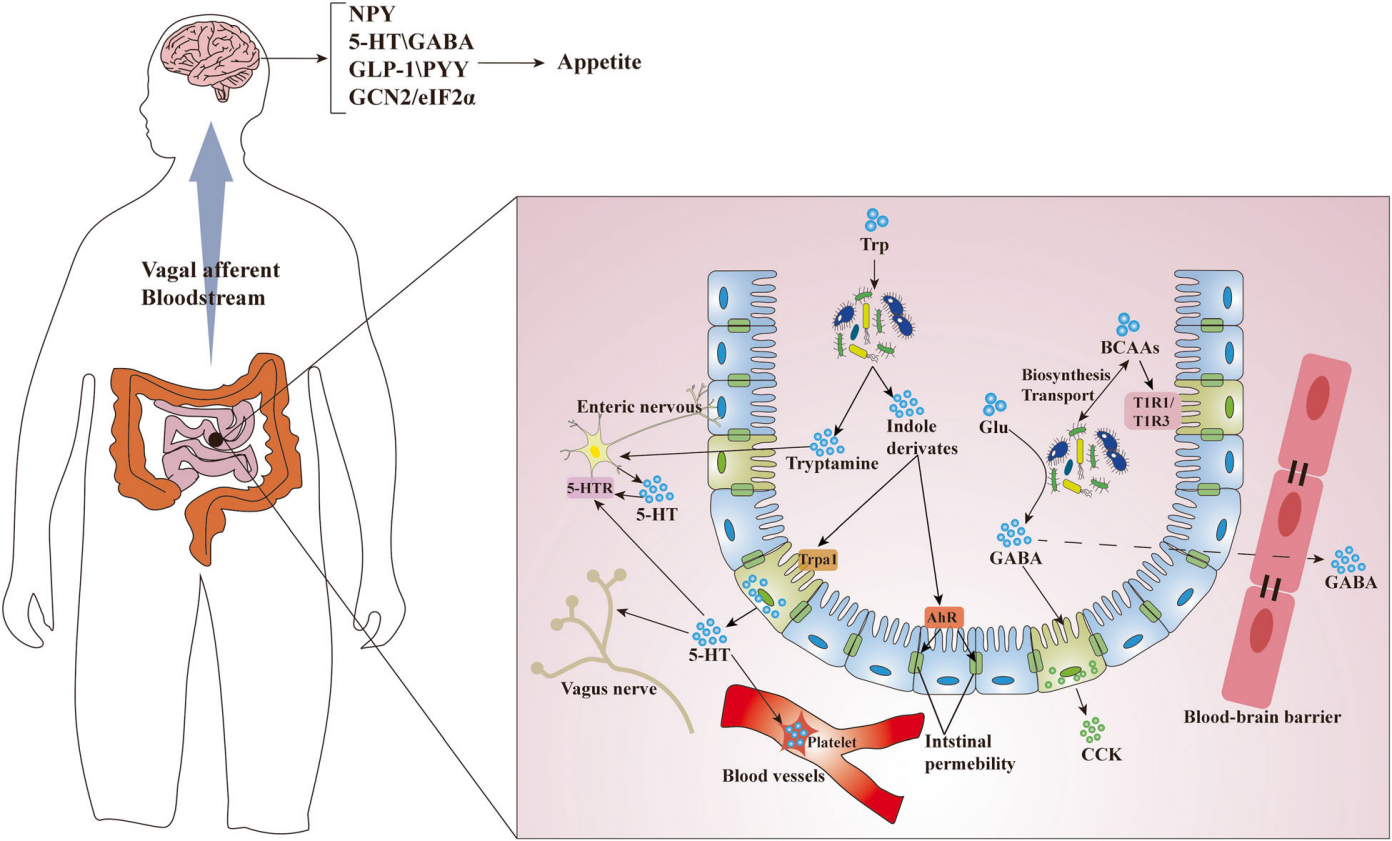
Gut microbial metabolites derived from amino acids influence host appetite control. Microbiota-derived amino acids mediate a variety of effects on appetite control. (1) Trp can be metabolized by commensal bacteria to produce tryptamine that affect the production and secretion of 5-HT, and some indole derivatives that are associated with maintaining intestinal permeability. 5-HT can act as neurotransmitter that conveys signals from the gut to the brain and mediate appetite control. (2) Glu can be metabolized by gut microbiota to produce GABA, which is considered a neurotransmitter to regulate the secretion of appetite-related hormones and intestinal motility. (3) Gut microbiota are involved in the biosynthesis and transport of BCAAs. The imbalance of BCAAs: non-BCAAs ratio can influence the 5-HT production in the hypothalamus. In addition, BCAAs can control appetite through mediating intestinal amino acid receptors and hypothalamic NCG2/eIF2α signaling. The sensory, hormonal, and neural signals are sent to the brain through vagal afferents or bloodstream to regulate appetite

**Fig. 3:**
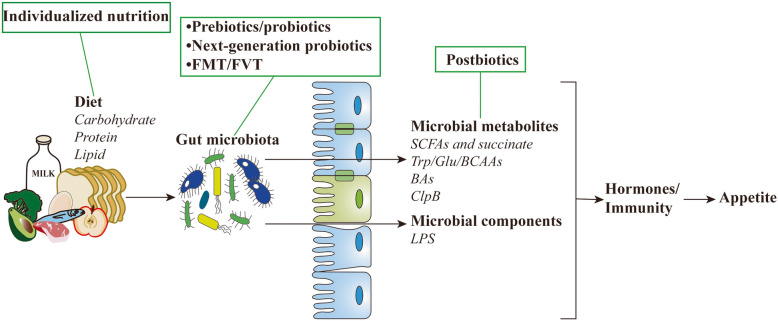
Potential clinical applications related to gut microbiota in appetite-related disorders. Gut microbial composition and metabolites contribute appetite control through altering the production and secretion of appetite-related hormones and influencing the immune system. Modulation of gut microbial composition is feasible via various strategies, including dietary interventions, probiotics, prebiotics, next-generation probiotics, FMT, and FVT. In addition, postbiotics can specifically and precisely change the microbial metabolites

In this review, we further discuss the most recent insights regarding how the gut microbiota and its metabolites that are implicated in food consumption may link to appetite-related hormonal and neural signals. Microbiota-derived metabolites and components are focused on the appetite regulation via modulating hormone secretion and immune system function (Figs. 1 and 2). van de Wouw et al. have reviewed mechanistic insights into the pathway of how gut microbial metabolite, including GABA, BAs, and SCFAs, may contribute to host metabolism and appetite [25]. Notably, microbial metabolites related to appetite control are not limited to these. We herein evaluate a series of recently discovered gut microbial metabolites, such as succinate, branched-chain amino acids (BCAAs), and bacterial proteins, and their potential role as messengers between the gut microbiota and host energy homeostasis in appetite control. Based on the importance of organic acids, amino acids, and fatty acids related to gut microbiota metabolism, we also provide insights for microbiome-targeted therapies to treat or prevent appetite-related disorders (Fig. 3).

## Gut microbiota and appetite-related hormones

The physiological control of appetite is mediated by circulating orexigenic and anorexigenic hormones (e.g., leptin, insulin, and ghrelin) produced by peripheral organs, including gut, adipose tissue, and pancreas. Here, we summarize the impacts of specific changes in the microbial compositions on appetite-related hormones, which play a key role in modulating brain behavior and function through the humoral or the neural pathway.

### Leptin

Leptin, secreted mostly from the white adipose tissues, reflects the body’s energy stores [26, 27]. The stomach and intestine are also sources of leptin and contain leptin receptors [28, 29]. Leptin can cross the blood-brain barrier (BBB) and then activates leptin receptors on the two subsets of neurons in the hypothalamic arcuate nucleus (ARC). Specifically, leptin can activate the anorexigenic neurons expressing proopiomelanocortin (POMC) and inhibit orexigenic neurons expressing neuropeptide tyrosine (NPY) and agouti-related protein (AgRP), which collectively inhibit host appetite [30–34].

Evidence from rodent experiments suggests that gut microbial abundance and richness are related to the leptin signaling. For example, in human with and without obesity, lower bacterial richness is associated with higher circulating leptin concentrations [35]. In addition, in vivo and in vitro studies showed that the translocation of living gut microbiota to adipose tissues induced by increased intestinal permeability influences energy metabolism through inhibiting the leptin signaling in obese humans and mice [36, 37]. Leptin treatment decreases the hypothalamic NPY and AgRP expression in germ free (GF) mice, whereas has no effect in WT mice [38], suggesting an important role of gut microbiota in leptin signaling. Furthermore, the depletion of gut microbiota inhibits leptin signaling and food intake in mice fed with the normal diet, whereas inhibits food intake but without affecting leptin signaling in mice fed with the high-fat diet [39, 40], demonstrating that the effect of gut microbiota on leptin signaling is dependent on the diet. Interestingly, probiotics or prebiotics supplementation has different and even contrary effects on the leptin signaling and food intake in genetically and diet-induced obese mice [41–44]. These conflicting results may be because the functional consequences of the microbial taxa shift have inconsistent outcomes to the leptin signaling [41], and the precise outcomes of specific microbiota need further investigations. Furthermore, whether the prebiotics and probiotics have comparable influence in animals and humans with eating disorders is still unclear.

### Ghrelin

Contrary to leptin, ghrelin is mainly a stomach-derived hunger hormone that acts as a ligand for the growth hormone secretagogue receptor (GSHR). In addition to the stomach, the fetal islets and adults’ duodenum also synthesize and secret ghrelin, but the quantity appears to be small [45, 46]. Ghrelin can transmit starvation signals to the brain via binding to its receptor on vagal afferent neurons [47]. Ghrelin is also able to cross the BBB and directly activate AgRP/NPY and inhibit POMC neurons through binding to GSHR in the brain, which will lead to increased food intake and decreased energy expenditure [48–51]. Furthermore, recent studies have shown that the gut microbiota are involved in regulating appetite through modulating ghrelin-related signaling pathways [52, 53]. For example, administration of prebiotics, such as inulin and oligofructose, inhibits feed intake via enhancing the synthesis of glucagon-like peptide (GLP-1) and peptide YY (PYY), as well as inhibiting the ghrelin production in obese and healthy adults [54, 55]. However, another intervention study with oligofructose-enriched inulin for 16 weeks in obese children decreased food intake and enhanced blood fasting ghrelin concentration, while has no significant effects on the GLP-1 and PYY, and insulin concentration [56]. The investigators suspected that increased ghrelin may act as a defense against diet-inhibited caloric intake. These conflicting experimental results call for studies in which the ghrelin signal and appetite are tested after prebiotics or probiotics interventions to explore the potentially dietary strategies for abnormal eating behavior treatment. In addition to metabolic needs, the hedonic effects of food can also induce food intake in individuals, which may be because eating food can make them feel better and relieve stress [57]. Studies have demonstrated that leptin and ghrelin are responsible for both homeostatic and hedonic aspects of feeding by regulating dopamine signaling [57–60]. Overall, these results show that the gut microbiota may regulate feed intake by regulating leptin-related signaling pathways.

### Insulin

In addition to controlling glucose and energy homeostasis, insulin can function as a satiety signal [61]. Various studies have shown that insulin-related signaling pathways are associated with decreased food intake in insects, mice, and humans [62, 63]. Similar to leptin and ghrelin, insulin can also cross the BBB and control appetite by acting on the POMC/CART and NPY/AgRP neurons after binding with its receptor [38, 62]. In addition, insulin and leptin treatment decrease food intake by increasing the expression of angiopoietin-like protein 14 (Angpt14) and inhibiting hypothalamic AMPK signaling in mice [64]. As demonstrated in humans and mice, insulin signaling is influenced by the gut microbial communities. For example, humans with low gut bacterial richness have higher insulin resistance [35], whereas mice with deficient and deleted gut microbiota have higher insulin sensitivity [65, 66]. Furthermore, altered gut microbiota induced by probiotics inhibits food intake by alleviating insulin resistance and inhibiting NPY expression in diet-induced obese mice [44, 67]. These observations suggest that the gut microbiota could participate in modulating appetite by influencing central insulin signal.

In summary, hormones derived from peripheral organs participate in various metabolism processes involved in appetite, such as energy homeostasis and hedonic feeding. It remains possible that altered gut microbiota may have an influence on host appetite through regulating the secretion of appetite-related hormones, pending confirmed evidence from more rigorous tests in clinical trials.

## Gut microbial metabolites and appetite

It has long been suggested that gut microbial metabolites play a key role in generating energy and mediating microbiota-gut-brain communication, which may affect the physiological and psychological functions of mammals [68, 69]. A better understanding of the interaction between gut microbial metabolites and appetite will help to design personalized nutritional strategies for treating eating disorders. We will hereafter update the molecular mechanisms, and we also reviewed some other microbial metabolites that are related to appetite control.

### SCFAs

The SCFAs (i.e., acetate, propionate, and butyrate) are generated by the gut bacterial fermentation of low-digestible polysaccharides, such as dietary fibers. In addition to providing energy, SCFAs widely act as signaling molecules and play a key role in appetite control. A piglet study observed both negative (*Ruminococcaceae* and *Lactobacillus*) and positive (*Prevotella*) relationships between the SCFAs and lactic acid-producing gut microbiota and feed intake [70]. SCFAs exhibit their metabolic and appetite-related functions by binding to the G-protein-coupled receptors in various tissues and organs, including free fatty acid receptor 3 (FFAR3, GRP41) and free fatty acid receptor 2 (FFAR2, GRP43). Signaling via these receptors has contrary effects (reviewed in detail elsewhere [71]). On one hand, SCFAs can activate ghrelin-related signaling and inhibit the insulin secretion by activating free fatty acid receptor 3 (FFAR3, GRP41) in islets, but the effects of SCFAs on appetite through activating GRP41 are unclear [71–73]. On the other hand, SCFAs can inhibit appetite by binding to the free fatty acid receptor 2 (FFAR2, GRP43), which further activates the release of GLP-1, PYY, insulin, and leptin to signal to appetite system (Fig. 1) [74–77]. GLP-1 and PYY, two anorexigenic hormones [48, 78–82], can cross the BBB and act as direct brain neuropeptides to activate POMC [49, 83]. In addition, GLP-1 and PYY help to increase insulin sensitivity and slow gastric emptying and intestinal motility to affect appetite [84–87]. Intriguingly, acute colonic propionate delivery decreases food intake and stimulates the secretion of PYY and GLP-1, while long-term colonic propionate delivery has little effects on PYY and GLP-1 release in humans [74], which may be associated with propionate resistance. Besides, a clinical study indicated that the reward processing and hedonic response rather than the secretion of PYY and GLP-1 contribute to the inulin-propionate-caused energy intake reduction [88]. Furthermore, gut-derived SCFAs entering the bloodstream can also cross the BBB and directly affect appetite-related neurons in the brain [89]. For example, intraperitoneal injection of acetate significantly decreases food intake by increasing the expression of POMC and inhibiting agouti-related peptide (AgRP) in the hypothalamus, but without affecting the concentration of circulating PYY and GLP-1, suggesting that acetate may directly regulate appetite by generating an anorectic signal in the hypothalamic ARC [89]. Moreover, findings in human and animal studies suggested that increased intestinal permeability, partially induced by microbial alterations, is associated with the eating disorders [90–95], which may be due to the elevated circulating ClpB and LPS levels [96–99]. A recent study showed that the FMT-enhanced SCFA levels contribute to the decreased intestinal permeability and increased food intake in mice with neurological disability [100]. Taken together, these results suggest that gut-derived SCFAs are involved in appetite control through hormonal and central effects. However, different dietary fiber supplementation has different influence on host appetite and energy intake, which is due to the complex effects of different SCFAs on energy metabolism (reviewed in detail elsewhere [25, 101]). These conflicting results suggest that further mechanistic research is needed to investigate the influences of each SCFA or in combination on appetite control in order to precisely and systematically explore the relationship between SCFAs and appetite control.

### Succinate

Succinate is a common product produced by gut microbial carbohydrate fermentation, which was not noticed in van de Wouw and co-workers’ review [25]. Evidence from human studies suggests that the microbiota-derived succinate contributes to the host energy homeostasis. The obese individuals display increased circulating succinate concentrations, which could be attributed to those with obesity produce more gut microbiota-derived succinate as compared with healthy individuals [102]. Meanwhile, in obese patients, dietary weight loss intervention alters gut microbiota and decreases circulating succinate concentration [102]. Nevertheless, the results from studies investigating the effects of succinate on appetite-related signaling are inconsistent. One series of studies suggested that succinate treatment improves glucose and insulin tolerance and elevates energy expenditure, but without affecting the food intake in mice fed with high-fat/high-sucrose and high-fat diet [103, 104]. Interestingly, another study demonstrated that supplementation with succinate reduces food intake and plasma insulin concentration in genetic obese (*ob/ob*) mice [105], which might be due to the succinate-promoted intestinal gluconeogenesis that can be detected by the hepatoportal glucose sensor and then send an anorectic signal to the brain [106]. Overall, these outcomes derived from mice models indicated that succinate might be involved in appetite control. However, studies in humans that investigate the effects of succinate on appetite regulation are currently lacking.

### Tryptophan (Trp)

The gut microbiota plays an important role in controlling the availability and metabolism of Trp, which directly or indirectly regulates metabolic homeostasis and even appetite [107–110]. Trp can both affect the gut hormone secretion and cross the BBB to directly activate satiety circuits in the brain [111]. Amounting studies have been performed to explore the effects of dietary supplementation and reduction of Trp on appetite control, whereas the results are inconsistent and contradictory. For example, animal studies showed that Trp administration can stimulate food intake by enhancing ghrelin, 5-HT, neuropeptide Y (NPY), and the pituitary growth hormone-insulin-like growth factor (GH-IGF) signaling [112, 113]. However, intragastric or intraduodenal Trp dose dependently inhibits appetite and promotes the production of CCK, GLP-1, and PYY in the healthy men, whereas failed to affect appetite in the obese men [114–116]. In line with this, an animal study also showed that the supplementation of 5% Trp increases satiety and reduces feed intake in healthy rats [117]. Moreover, severe Trp restriction decreases the plasma leptin and ghrelin concentrations and increases the plasma GLP-1 and PYY concentrations, which leads to decreased feed intake and body weight, while moderate Trp restriction increases energy expenditure in obesity-prone rats [118]. These results suggest that the effects of Trp on appetite are complex and depend on the dosage of dietary Trp and the host metabolic conditions.

In terms of metabolites produced by the gut microbiota, indole can serve as a molecular signal to regulate food intake and appetite by stimulating GLP-1 secretion in enteroendocrine L cells [119–121]. A recent study found that the derivatives of indole, including indole-3-ethanol (IEt), indole-3-pyruvate (IPyA), and indole-3-aldehyde (I3A), also can decrease intestinal permeability by binding to their receptor, ary hydrocarbon receptor (AhR) [122], which may contribute to the intestinal barrier function and appetite control. Moreover, Trp is the precursor of 5-HT and dominates the synthesis rate of intestinal and central 5-HT [108, 123, 124]. Studies using GF mice, gnotobiotic mice recolonized with spore-forming microbiota from SPF mice, and healthy humans showed that the gut microbiota participates in the 5-HT production and influences the serotonergic neuronal networks [125, 126]. Mechanistically, some studies have revealed that gut microbiota-derived SCFAs increase the circulating 5-HT concentration via promoting tryptophan hydroxylase (Tph) 1 transcription in ECs [126, 127], whereas Martin et al. reported that acetate or butyrate treatment fails to affect the 5-HT secretion in duodenal and colonic ECs [128]. Additionally, a recent study showed that indole and its derivate, indole-3-carboxaldehyde (IA1d), produced by *Edwardsiella tarda*, form Trp that can bind with transient receptor potential ankyrin Aa (Trpa1) to enhance 5-HT secretion from enteroendocrine cells, and in turn stimulates intestinal motility and regulates CNS function in both human and mouse models [129]. Moreover, other microbial metabolites, such as deoxycholate, α-tocopherol, p-aminobenzoate, and tyramine, also can stimulate the 5-HT secretion from ECs and enteric neurons [122, 126, 130]. Approximately 90% of the circulating 5-HT is produced from enterochromaffin cells (ECs) in the host gut and then stored in circulating platelets that convey 5-HT into every organ and tissue, including the brain [126]. Thus, although 5-HT cannot cross the BBB directly, platelet-derived 5-HT is able to increase the level of CNS 5-HT, which might link the intestinal 5-HT with brain function [131]. Various studies have shown that 5-HT plays a vital role in regulating energy metabolism and suppressing appetite through various mechanisms, including improving insulin sensitivity and mediating intestinal functions (i.e., motility, secretion, absorption, and sensory) by directly acting on the enteric nervous system and hypothalamic AgRP and POMC neurons [132–139]. Thus, it is clear that the gut microbiota can involve in appetite control by modulating intestinal and central 5-HT signaling.

In summary, gut microbial regulation of Trp metabolism involves in host appetite control (Fig. 2), although these effects are somewhat inconsistent and the reasons remain unclear, but might be partially due to whether the stimulation of Trp reach the “threshold” required to regulate appetite and energy intake. These findings encourage the future investigation of the specific role and mechanism of Trp and its metabolites derived from gut microbiota in appetite control.

### GABA

GABA is a microbial metabolite from dietary glutamate and acts as a neurotransmitter that contributes to the communication between the gastrointestinal tract and brain [140]. Most *Lactobacilli* and *Bifidobacteria* strains have the *Gad* genes that can encode glutamate decarboxylase to synthesize GABA [141, 142]. Obese patients have decreased abundance of glutamate-fermenting microbiota, as well as increased circulating glutamate level [143], indicating that the gut microbiota participates in host energy hemostasis through modulating glutamate metabolism. This hypothesis is further confirmed by studies using GF, gnotobiotic, and humanized mice [144–146].

GABA is strongly associated with appetite control as the disruption of GABA signaling pathways can inhibit postweaning feeding, blunted NPY-induced hyperphagia, and hunger-induced appetite [147, 148]. Mechanistically, GABA has long been known as a molecular signal involved in modulating the gastrointestinal motility and the secretion of appetite-related hormones (extensively reviewed elsewhere [149]). In addition, GABA functions as an inhibitory neurotransmitter in the CNS. GABA is required, at least in part, to the activation of AgRP neurons [150, 151]. It is noteworthy that due to the variation of chemicals and compounds and the different administration methods, the data on whether GABA can cross the BBB is inconsistent [152–154]. In addition, the majority of studies demonstrating the effects of GABA on host health focus on dietary GABA rather than host endogenic GABA. The rumen-protected GABA supplementation increases feed intake and inhibits CCK signaling in growing lambs and cows [155, 156], which may be because GABA is co-expressed and shares the similar signal transduction pathways with CCK [150, 156, 157]. Thus, it does appear to be reasonable to hypothesize that GABA might be involved in the appetite control via acting on its receptors in the gastrointestinal tract and brain, which in turn influences the secretion of gut hormones and activates central neurons, respectively. However, the research on the relationship between the gut microbial-derived GABA and appetite control is limited; thus, further studies are needed to investigate the role of GABA produced by the gut microbiota on host metabolic health and determine whether GABA can cross the BBB and act on the CNS to regulate appetite.

### BCAAs

BCAAs, including leucine, isoleucine, and valine, are derived from the diet as well as can be de novo by the gut microbiota. The gut microbiota exhibits enriched genes related to BCAA biosynthesis (*Prevotella copri* and *Bacteroides vulgatus*), degradation (*Bacteroides thetaiotaomicron* and *Dorea longicatena*), and uptake (*Butyrivibrio crossotus* and *Eubacterium siraeum*) [158, 159]. Human and animal studies revealed the relationship between gut microbiota, circulating BCAA level, and insulin resistance [160–164]. There is also growing evidence that demonstrates that dietary supplementation or reduction of BCAAs induces alterations in host appetite, yielding inconsistent results. For example, long-term BCAA supplementation decreases feed intake in high-fat diet-induced obese rats [165]. Diet containing high ratio of BCAAs to other AAs (non-BCAAs) induces hyperphagia in mice, which might be because high BCAAs: non-BCAAs intake downregulates the synthesis of central 5-HT [166]. Our recent study also found that supplementation of BCAAs to low-protein diet increases the relative abundance of colonic *Lactobacillales* and promotes food intake in piglets [167]. Furthermore, in another study using piglets, long-term dietary deficiency of BCAAs inhibits food intake which might be associated with the enhanced expression of intestinal amino acid receptors, type-1 taste receptors 1 (T1R1) and type-1 taste receptors T1R3, that can activate the CCK secretion and the enhanced hypothalamic GCN2-Eif2α signaling that is involved in the energy metabolism and inhibiting appetite [168]. We speculate that the inconsistent results might be due in part to whether the studies involve the manipulation of the balance between the BCAAs and non-BCAAs. Although, the mechanism by which BCAAs involve in appetite control are complex and controversial (Fig. 2), these data suggest that targeting at gut microbiota for maintaining amino acid metabolism and homeostasis might be crucial for improving appetite control.

### BAs

BAs are synthesized in the liver and released into the gastrointestinal tract and are involved in intestinal absorption of lipid, as well as metabolic and inflammatory signaling pathways [169]. Previous studies have shown that the gut microbiota plays a crucial role in the BA metabolism by deconjugation, dehydrogenation, and dihydroxylation of primary BAs [170–172]. The synthesis of BAs mainly depends on cholesterol 7α-hydroxylase (CYP7A1) and sterol-27-hydroxylase (CYP27A1) that are regulated by the gut microbiota [173, 174]. Moreover, BAs have been reported to modulate appetite by directly binding with their receptors in the gastrointestinal tract to regulate the secretion of appetite-associated hormones. For example, altered BA composition enhances the GLP-1 and PYY secretion from enteroendocrine cells via activating GRP119 and Takeda G-protein-coupled bile acid receptor (TGR5) in ECs, which in turn slows gastric emptying and ultimately decreases food intake in mice [175, 176]. Collectively, it is reasonable to conclude that BA metabolism, which is greatly affected by the gut microbiota, can mediate appetite regulation by modulating appetite-related hormones.

## Gut bacterial proteins

The gut microbiota, including the bacteria, fungi, virus, and archaea, can produce identical protein sequences with appetite-regulating peptides (i.e., leptin, PYY, ghrelin, α-MSH, NPY, AgRp) [177]. ClpB, the best studied bacterial protein, can act as a mimetic of alpha-melanocyte-stimulating hormone (α-MSH) to result in similar anorexigenic effects [178]. Briefly, ClpB derived from *Escherichia coli* (*E. coli*) is capable of displaying the α-MSH-like function, such as enhancing PYY and GLP-1 secretion, and directly activating anorexigenic neurons, and subsequently inducing satiety [13, 48]. An in vitro study showed that protein supplementation stimulates the secretion of ClpB from *E. coli*, which can induce satiety signaling by enhancing the PYY production in intestinal mucosal cells [179]. This observation was translated to rats treated with protein produced by *E. coli* showing inhibited host appetite, increased circulating GLP-1 and PYY concentrations, and activated hypothalamic POMC neurons, which may be due to the anorexigenic functions of ClpB [13]. In addition, a recent study revealed that food restriction increases plasma ClpB levels, which is associated with the increased relative abundance of *Enterobacteriaceae and* intestinal permeability, and in turn increases satiety by activating anorexigenic neurons in mice [96]. Altogether, these data support the possible mechanistic links between gut microbiota-derived ClpB and host appetite. Whether other specific gut microbial communities also can produce ClpB and contribute to appetite control is unknown.

The gut microbiota play a key role in regulating the immunoglobulin (Ig) production (reviewed in detail elsewhere [180]). Igs can react with α-MSH and then activate MC4R to involve in appetite control, which can be diminished by ClpB through neutralizing IgG [180, 181]. IgG has been reported to involve in controlling appetite by modulating leptin and ghrelin signaling pathways [182, 183]. Co-administration of ghrelin together with IgG from obese patients and ob/ob mice increased food intake in mice, which might be due to the inhibited ghrelin degradation induced by ghrelin-reactive IgG [183]. Another study revealed that the levels of plasma IgG and α-MSH were lower in rats with methotrexate-induced intestinal inflammation and anorexia, while anti-α-MSH IgG supplementation led to an attenuation of feed intake [184]. Besides, AN patients show higher levels of α-MSH-reactive IgM and α-MSH-IgG complexes that can bind and activate MC4R with a lower threshold than α-MSH alone than normal controls [185, 186]. Additionally, a recent study revealed that the activation of the mechanistic target of rapamycin complex 1 (mTORC1) signaling can modulate IgA secretion, which contributes to decreased *Lactobacillus johnsonii* Q1-7 abundance and inhibited food intake in mice [187]. These data suggest a link between the gut microbiota, autoimmune system, and appetite control. These bacterial proteins could be used as biomarkers of eating disorders but needs further confirmation.

## Clinical relevance

Abnormal regulation of appetite can cause eating disorders and obesity [188–190], which are severe and life-threating mental illness. In a study of Australian adolescents, 22.2% of participants suffered from eating disorders [191]. AN, BN, and binge eating disorders (BED) are the three most common eating disorder diagnoses. The most common explanation for AN is the constant fear of becoming overweight and disturbed cognitions about body perception [192]. Individuals with AN show severe underweight, and other psychiatric complications include depression and anxiety [7, 193]. An increasing number of studies indicate that individuals with BN and binge eating disorders have higher incidence rates of obesity and related metabolic diseases, such as type 2 diabetes and cardiovascular disease compared to individuals with no history of eating disorders [194–196].

In recent years, there has been keen interest in exploring the gut-microbiota-brain axis [197–201]. Growing evidence suggests that the gut microbiota can act as an effective regulator of host body weight and psychiatric disorders [140, 202–205]. Fecal microbiota transplantation (FMT) from a healthy individual to an AN patient has led to weight gain by increasing the production of SCFAs and composition of beneficial microbiota [69]. Besides, gut viral community contains mostly phages, which can infect bacteria and lead to cell lysis [206]. Similar to FMT, a recent study has mounted that fecal virome transplantation (FVT) also has therapeutic potential against metabolic diseases, including obesity and T2D [207]. Another series of studies demonstrated that administration of prebiotics and probiotics have the ability to regulate food intake and ameliorate obesity and associated disorders in humans and experimental animals [208–210] (Table 1). However, the effects of probiotics on appetite-related hormones and appetite in obese and overweight subjects are inconsistent, which has been reviewed elsewhere recently [211]. Thus, future studies with high methodological quality and low risk of bias are needed to determine precise the effects of probiotics on appetite regulation. In addition to the common probiotics (mainly include *Lactobacillus* spp. and *Bifidobacterium* spp.), the next-generation probiotics (i.e. *Akkermansia muciniphila*, *Bacteroides thetaiotaomicron*, *Bacteroides vulgatus*, *Faecalibacterium prausnitzii*, *Ruminococcus bromii*, and *Roseburia*) have been gradually identified and considered to have the potential for treating metabolic diseases due to the development of culturing methodologies and genome and metagenome sequencing techniques [93, 212–217]. Despite the technologies limit the use of the new identified probiotics, it may provide opportunities to use dietary interventions, such as prebiotics, to treat appetite-related disorders via modulating specific gut microbiota [218]. In a recent study, Ortega-Vega et al. found that the gut microbial diversity and some specific gut microbiota with heritability are associated with the variants in these genes encoding ghrelin, MC4R, GLP-1, NPY, and PYY and metabolic diseases, revealing that, to some extent, the intricate links between host genetics and gut microbiota are related to appetite modulation, which expands our understanding of the functional attributes of the gut microbiome in metabolic and eating disorders as well as open new therapeutic manipulation of specific microbiota [219].

**Table 1:** Studies on probiotics/prebiotics and appetite control in human and animals

	Human/animal	Effects	References
Probiotics			
*Lactobacillus paracasei*	Men	Decreased food intake	[220]
*Lactobacillus acidophilus*, *Bifidobacterium bifidum*, *Bifidobacterium lactis*, *Bifidobacterium longum*, *Lactobacillus rhamnosus*, *Lactobacillus reuteri*, magnesium stearate, and maltodextrin	Women	Decreased hunger score and emotional eating score by inhibiting NPY	[221]
*Lactobacillus acidophilus and Lactobacillus casei*	Broiler chicken	Decreased feed intake	[222]
*Hafnia alvei HA4597*	High-fat-diet-fed obese mice	Decreased feed intake by increasing ClpB production	[223]
*Lactobacillus brevis SBC883*	Rats	Increasing feed intake by increasing serotonin and ghrelin production	[224]
*Lactobacillus casei*	Children with diarrhea	Improved appetite by altering gut microbiota (i.e. increased *Bifidobacteria* and *Lactobacillus*)	[225]
*Lactobacillus rhamnosus*	Larvae	Increased feed intake by altering gut microbiota and neuropeptide production	[226]
*Lactobacillus rhamnosus*, *L. acidophilus*, and *Bifidobacterium bifidum*	Diet-induced obese mice	Decreased feed intake by altering gut microbiota and decreasing intestinal permeability	[227]
*Lactobacillus rhamnosus*	Obese women	Decreased desire to eat	[228]
*Lactobacillus rhamnosus*	Zebrafish	Decreased appetite by altering gut microbiota	[229]
Prebiotics			
Oligofructose-enriched inulin	Children with overweight/obesity	Decreased food intake by decreasing ghrelin and increasing PYY	[56]
Inulin-type fructans and/or whey protein	Adults with overweight/obesity	Decreased hunger, desire to eat, and prospective food consumption by altering gut microbiota (i.e., increased *Bifidobacterium*)	[230]
Chicory	Mice	Induced satiety by altering gut microbiota (i.e., *Firmicutes/Bacteroidetes* ratio, *Alloprevotella*, *Blautia*) and increasing CCK and GLP-1	[231]
Mannose oligosaccharide	Diet-induced obese mice	Suppressed appetite by altering gut microbiota (i.e., increased *Bifidobacterium* and *Lactobacillus*) and increased SCFAs production	[232]
Digestion-resistant maltodextrin/fructooligosaccharides	Diet-induced obese rats	Decreased energy intake by increasing GLP-1 production	[233]

Although there is as yet no evidence that such treatments would be safe and efficient for feeding-related diseases, these studies provide proof of concept for microbial interventions in directly or indirectly counteracting eating disorders (Fig. 3). While the mechanism by which how the gut microbiota may regulate eating behavior is still elusive, efforts to alter the commensal microbiota by administration of probiotic, prebiotic, phage, and even FMT highlight the potential of microbiota interventions in treating eating disorders by modulating host appetite and reducing food-related and body-related fears; the potential of microbiota as a modifier of metabolic disorders induced by abnormal appetite control; and the potential of microbial amelioration of psychiatric diseases such as depression and anxiety caused by eating disorders.

## Conclusions

In this review, the direct and indirect molecular mechanisms how the gut microbiota regulates host appetite were summarized. Although a great number of studies have already linked the gut microbiota and eating behavior, the precise mechanisms through which the gut microbiota influence particular eating disorders, such as anorexia nervosa and food addiction, have not yet been fully deciphered. Understanding how some specific members of the gut microbiota are involved in appetite control may be important to develop novel preventive and therapeutic interventions and even prediction for eating disorders. It should be noted that it is extremely difficult to define the optimal gut microbiota, since individuals have different gut microbiota composition, and even in the same host, the gut microbiota have complex variations and evolutions during the whole life cycle due to various diets, environments, genes’ expression, and so on [234–239]. The gut microbiota could be the so-called healthy microbiota as long as it benefits the individual who harbors it. Thus, further efforts should be made to explore the dynamics and effects of gut microbiota changes and differences, in order to design microbiota-based therapeutic strategies for different individuals during different life stages. To date, our understanding of the gut microbiota roles in modulating appetite is mainly based on in vitro studies and rodent models. With regard to this, it will be essential to conduct well-designed clinical trials or assemble clinical data, in order to fill the large gaps between clinical and experimental knowledge and translate the proof of concept acquired from animal models to the clinical setting. Consequently, these studies may potentially be applied for probiotics, prebiotics applications, and FMT, as an effective treatment for eating-related diseases in the future.

## Data Availability

Data sharing not applicable to this article as no datasets were generated or analyzed during the current study.

## Abbreviations

AN: Anorexia nervosa
BN: Bulimia nervosa
SCFAs: Short-chain fatty acids
GRP43: Free fatty acid receptor 2
GRP41: Free fatty acid receptor 3
GLP-1: Glucagon-like peptide
PYY: peptide YY
POMC: Pro-opiomelanocortin
MSH: Melanocyte-stimulating hormone
MC4R: Melanocortin-4 receptor
GABA: γ-aminobutyric acid
AgRP: Agouti-related peptide
ARC: Arcuate nucleus
Trp: Tryptophan
5-HT: Serotonin, 5-hydroxytryptamine
NPY: Neuropeptide Y
GH-IGF: Growth hormone-insulin-like growth factor
CCK: Cholecystokinin
BAs: Bile acids
GF: Germ-free
FXR: Farnesoid X receptor
TGR5: Takeda G-protein-coupled bile acid receptor
CART: Amphetamine-regulated transcript
WT: Wild-type
Angpt14: Angiopoietin-like protein 14
ClpB: Caseinolytic protease
TLRs: Toll-like receptors
PAMPs: Pathogen-associated molecular patterns
LPS: Lipopolysaccharide
Igs: Immunoglobulins
mTORC1: Mtormechanistic target of rapamycin complex 1
ABA: Activity-based anorexia
